# Single-Layer Metasurface-Based Reflectarray Antenna with H-Shaped Slotted Patch for X-Band Communication

**DOI:** 10.3390/nano14181495

**Published:** 2024-09-14

**Authors:** Jawad Ali, Ashfaq Ahmad, Dong-you Choi

**Affiliations:** Communication and Wave Propagation Laboratory, Department of Information and Communication Engineering, Chosun University, Gwangju 61452, Republic of Korea; j.ali@chosun.ac.kr (J.A.); ashfaquetb11@gmail.com (A.A.)

**Keywords:** reflectarray antenna, H-shaped slotted patch, FEBI, high gain, reflection phase range

## Abstract

In this study, a metasurface-based reflectarray is designed for X-band applications. The unit cells are equipped with an H-shaped slotted patch for additional resonance and phase range. Linear phase variation by altering the length of the patch is realized with a range exceeding $480^{\circ }$. The reflectarray is designed and fabricated on a thin and high-quality Rogers 5880 substrate. The Finite Element Boundary Integral (FEBI) method is used to simulate a 23×23 element reflectarray and then fabricated to achieve the measured results using an anechoic chamber. The peak gain of the proposed reflectarray is 25.5 dBi recorded with an aperture efficiency of 63.7% at a center frequency of 10 GHz. The cross-polarization and side-lobe levels in the entire band are less than −33 dB and −21 dB, respectively. Moreover, the proposed reflectarray antenna achieves a 20% 1-dB gain bandwidth.

## 1. Introduction

In this era of ultra-fast long-distance communication, microstrip reflectarray antennas [1] have gained popularity owing to their appealing low-profile feature communication, easy deployment, and amenability to print technology [2,3]. They have applications in medical technology ranging from smart health care to body-centric solutions, operating across multiple frequency bands [4,5,6]. They exhibit characteristics of planar-phase arrays and traditional reflector antennas. The phase response of the elements fabricated on a flat surface is responsible for the generation of a focused or shaped beam when illuminated by a feed [7,8]. Reflectarrays are deemed the best solution in terms of compatibility and affordability; however, these structures have a relatively narrower bandwidth than planar arrays and reflectors [9,10]. This limitation is due to the inherent narrow bandwidth behavior of the microstrip elements and the differential phase delay resulting from the different path lengths between the feed to each element [1].

Several techniques have been proposed to improve the bandwidth of reflectarray antennas. In a multilayer configuration, layered patches of varying sizes were used in [11,12]. Aperture-coupled patches with phase-delay lines are utilized in larger reflectarrays [13]. However, multilayer configurations are prone to increased weight, elevated fabrication costs, and complexity. Therefore, a single-layer reflectarray is more cost-effective and mitigates the alignment errors found in multilayer phase shifting elements [14]. Additionally, the miniaturization of reflectarrays has been proposed using metamaterials, particularly metasurfaces [15,16]. A subwavelength element [17,18] or multi-resonant element [19,20] exhibited a broad bandwidth in a single layer. In a previous study [13] for a broad 1-dB gain bandwidth, coupled aperture phase delay lines were used on various layers of the patches, whereas in other studies [21,22] they were connected to the elements in the same layer. The concepts of polarization-rotating elements and dielectric metamaterial-based broadband designs were presented in previous literature [23,24]. Different phase synthesis methods have also been used to obtain a broad bandwidth [19,25].

Although the above-mentioned single-layer reflectarrays exhibited broadband behavior, they still have some unfavorable traits. In [26], a thick substrate was utilized to achieve a $360^{\circ }$ phase range using a broadband reflectarray based on a sub-wavelength element. Broadband reflectarrays employing multi-resonant and polarization-rotating elements possess complex geometrical arrangements, necessitating time-intensive computational modeling and simulations of individual elements to ascertain their optimal outcomes. Similarly, existing techniques for phase accumulation in a single layer reduce the maximum gain for an enhanced bandwidth. Reconfigurable reflective and programmable metasurfaces have emerged as a solution for beam scanning, scattering manipulation, and polarization conversion with low cost and easy fabrication [14,27,28]. However, such metasurfaces have rarely been reported for a broad 1-dB gain bandwidth and still require time to evolve [29].

Similarly, different unit cells are introduced to attain high gain with broad linear phase variation. Minkowski-type resonating patches operating in X-band are presented in [30]. A phase range of more than $360^{\circ }$ with −18 dB side lobe levels is achieved using this element. In [31], an E-shaped polystyrene material was reported in which the arm dimensions are varied to obtain a phase variation of $360^{\circ }$. The gap between the arms controls the side lobe levels. To achieve high gain, a broadband stub-loaded reflectarray antenna is designed in [32]. This design configuration offers about a $600^{\circ }$ phase range with an aperture efficiency of 39%. In [33], another broadband reflectarray antenna is proposed consisting of circular rings, which offer a 24 dBi gain with side lobe levels less than −17 dB. The spatial phase delay in the reflectarray is compensated by delay lines that are connected to the microstrip patches. The phase delay lines also decrease the fabrication sensitivity along with the wide phase range.

In this paper, a novel metasurface-based H-shaped slotted (HSS) patch single-layer element with a large phase range and bandwidth is proposed for X-band applications. By adding an HSS, a broadband phasing element offering a range of more than $360^{\circ }$ is achieved using a narrow-band patch. As compared to the existing single-layer reflectarrays [34,35], the HSS offers a wide bandwidth and high gain. The HSS slotted patch element delivers a smooth transition of phase as the length changes, which is beneficial in achieving a progressive phase along the reflectarray panel. The proposed design also extends the current state-of-the-art slotted patch technology to be used in satellite communication in the X-band [29]. A center-fed reflectarray consisting of 529 unit cells with $0.3\lambda_{o}$ spacing at 10 GHz is designed and simulated using an HSS patch element. The reflectarray offers a maximum gain of 25.5 dBi with 63.7% aperture efficiency and 20% 1-dB gain bandwidth. This is in contrast to broadband designs [13,21,29]. The use of air layers for phase linearity in a reflectarray is reported in [36,37], but they introduce complications in terms of limiting bandwidth, the disruption of wave propagation, and alignment issues [29,38]. So, to overcome these issues and enhance our broadband design, an additional air layer was not added, and we achieved a smooth phase range without it. The cross-polarization and side lobe levels (SLLs) are significantly lower relative to those reported in other studies [35,39,40].

The remainder of this paper is organized as follows: Section 2 presents the unit cell design and simulation setup. Section 3 describes the reflectarray design and simulation. Section 4 discusses the measured results, and Section 5 presents the conclusion.

## 2. Design and Analysis of Unit Cell

**Figure 1:**
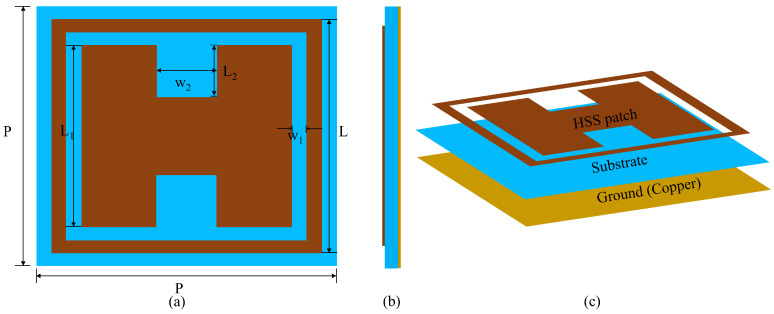
The schematics of the unit cell used in the reflectarray design. (**a**) Parametric depiction of the unit cell; (**b**) Side-view of the unit cell; (**c**) Different layers of the unit cell.

A schematic of the proposed wide-band reflectarray unit cell is shown in Figure 1. The HSS patch (copper) is designed and simulated on a Rogers 5880 $\left(\epsilon_{r}=2.2,tan\delta =0.0009\right)$ substrate with a thickness of 1.575 mm and a full ground layer (copper). With a low dielectric constant and low tangent loss, Rogers 5880 ensures minimal losses as compared to FR4 and RT/Duriod 6006, as they have dielectric constants 4.4 and 6.15, which are less suitable in terms of signal integrity. Similarly, Rogers 5880 is cost-effective and offers less fabrication complexity as compared to FR4 and RT/Duriod 6006.

**Figure 2:**
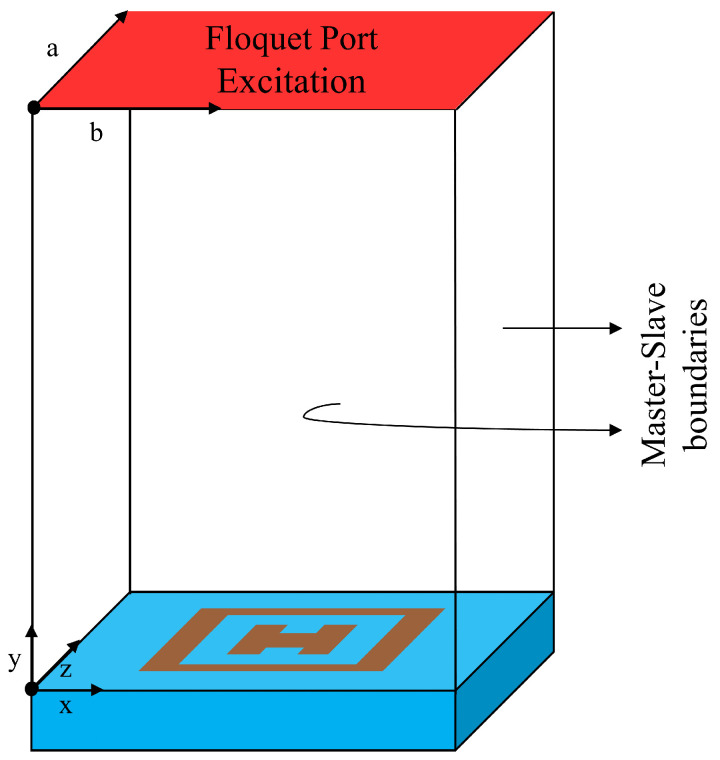
Simulation model in HFSS using master–slave boundary conditions.

To obtain an improved 1 dB gain and minimize the grating lobes [26], the periodicity (P) of the unit cell is chosen to be $0.3\lambda_{o}$ where $\lambda_{o}$ is the free space wavelength at a center frequency of 10 GHz. The outer lengths (L) of the HSS patch are one-to-one related to the inner lengths $L_{1}$ and the length and width of the separation $L_{2},w_{2}$ in the HSS patch. It should be noted that the separation within the HSS patch is in the shape of a square; thus, $L_{2}=w_{2}$. A comprehensive parametric study based on the lengths of the patch $\left(L,L_{1}\right)$, periodicity (P), and spacing between $\left(w_{1},w_{2}\right)$ to analyze the parallel phase, linearity, and range of the phase for a wide band is presented in this section, with details provided in Table 1. The design and simulation of the element are performed in HFSS at 10 GHz, and Floquet mode excitation with a master–slave boundary condition is used to investigate the phasing element, as shown in Figure 2. The simulation is performed on a rectangular wave-guide with the front and back as perfect electrical conductors, and the left and right surfaces are set as perfect magnetic conducting walls under normal incidence.

**Figure 3:**
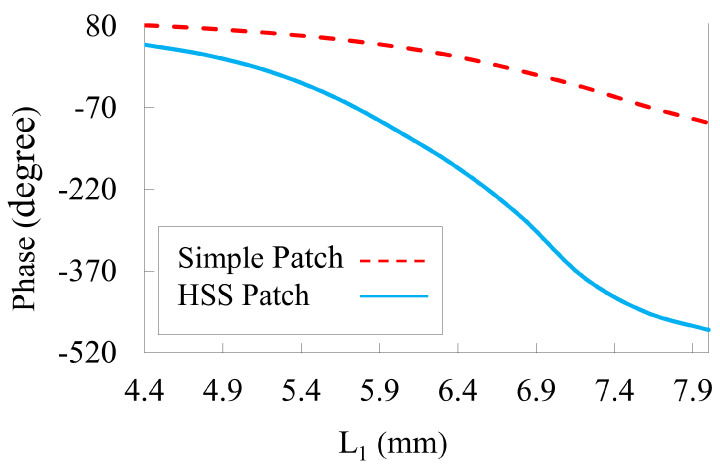
The phase of the unit cell with the length $L_{1}$ in comparison with a simple patch.

Initially, a unit cell is simulated with P = 9 mm and L = 8.4 mm to understand the working mechanism of the proposed structure. The HSS patch is derived from a simple square patch. Figure 3 illustrates the reflection phase of a square patch compared with that of a slotted patch as a function of the length of the slotted patch $\left(L_{1}\right)$ under normal incidence at 10 GHz. A phase range greater than $480^{\circ }$ is obtained by creating an HSS in the square patch. The HSS is responsible for the extra phase accumulation when the y-polarized wave is incident at 10 GHz and is responsible for providing a phase variation of more than $480^{\circ }$. The phase linearity is improved when both degrees of freedom are available, which helps in attaining low phase error distribution on the reflectarray aperture. A linear phase range is achieved, which is beneficial in terms of a minimized distribution of the phase error on the reflectarray aperture.

**Figure 4:**
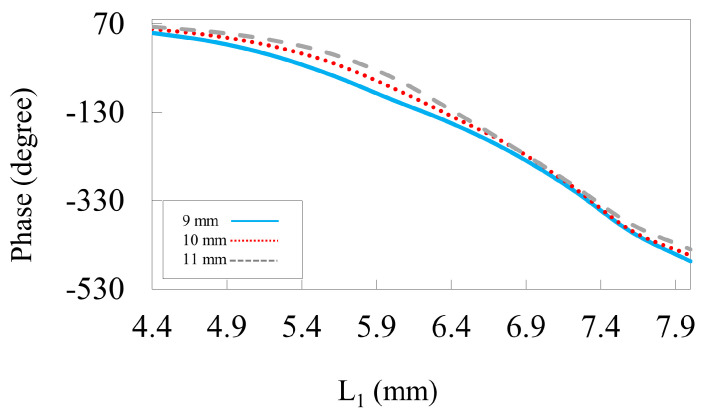
The reflected phase curve against the length $L_{1}$ at 10 GHz for different periodicities.

**Figure 5:**
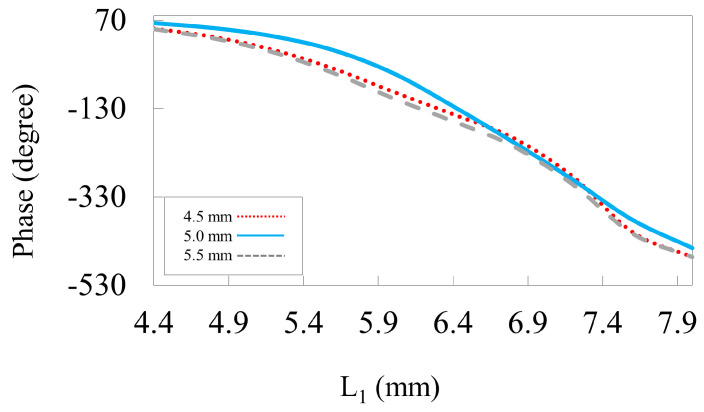
The reflected phase curve against the length $L_{1}$ for different spacings in the HSS patch.

The effect of grid spacing on the reflection phase is shown in Figure 4. The periodicity of the unit cell is crucial in determining the gain and overall efficiency of the reflectarray. To improve phase linearity, sub-wavelength elements should be employed. This behavior can be seen in Figure 4, in which reducing P from 11 to 9 mm results in a linear phase without changing the phase range. However, decreasing the periodicity affects the phase span of the unit cell. Therefore, for satisfactory behavior, the periodicity is taken as 9 mm, i.e., $0.3\lambda_{o}$ at 10 GHz. Figure 5 illustrates the spacing within the HSS patch using different values $\left(w_{1}\right)$. The inner width of the patch is crucial for the resonating element as this can significantly influence the phase response and resonance frequency, ultimately affecting the performance of the reflectarray unit cell. In our design, different values for $w_{1}$ are taken into account to maximize phase accumulation to achieve better phase compensation on the reflectarray plane, as shown in Figure 5. This suggests that a linear phase response is achieved for a spacing of $(w_{1}=$ 0.5 mm) rather than with low and high values.

**Figure 6:**
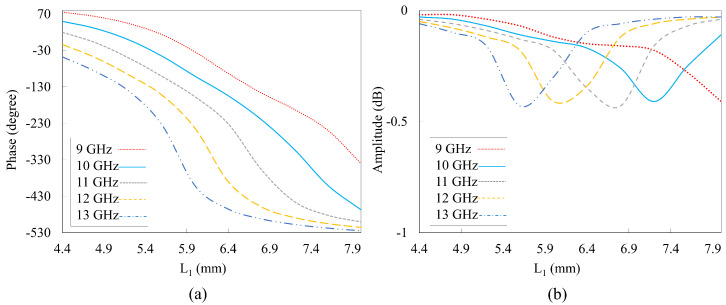
Reflected phase curve against the length $L_{1}$. (**a**) Reflection phase with changing frequencies. (**b**) Amplitude at changing frequencies.

The phase response of the designed unit cell with the selected frequency range of 9–13 GHz is shown in Figure 6a. Linear phase responses were observed across a range of frequencies, demonstrating the consistency and stability of the phase behavior in the HSS unit cell. Additionally, the phase responses at different frequencies are nearly parallel to each other, which is a clear indication of the broadband operation of the unit cell. This suggests that the unit cell maintains a uniform phase gradient over a wide frequency band, enhancing its performance in broadband applications. The ability to achieve such linear and consistent phase responses across multiple frequencies is crucial for ensuring effective and predictable beam steering in reflectarray designs. The variation in the amplitude for the frequency range is plotted in Figure 6b. It is shown that very low losses are recorded using the proposed phasing element over the broad frequency range. This indicates that the proposed HSS-based reflectarray bounces back most of the incident waves, which is beneficial in terms of operations. By moving toward a high frequency, the loss increases and a high loss is recorded at the second resonance. A maximum reflection loss of 0.43 dB is achieved at 13 GHz under normal incidence.

## 3. Reflectarray Design and Simulation

**Figure 7:**
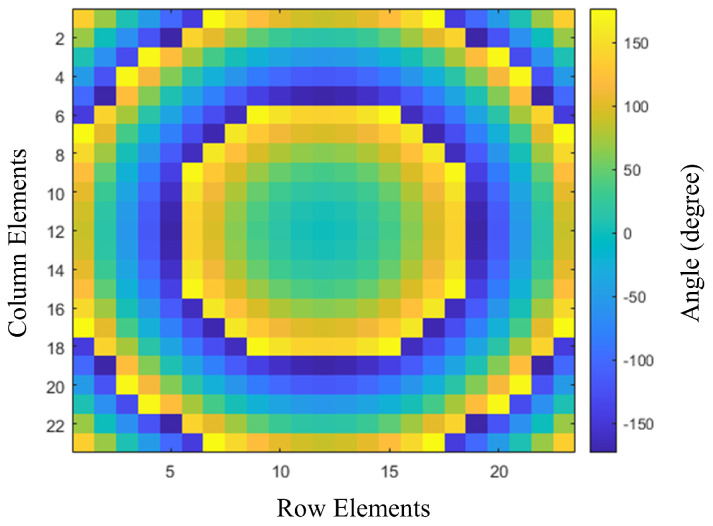
Phase shift distribution on the reflectarray panel with the angle of reflection.

The HSS elements are used to design a center-fed reflectarray on a Rogers 5880 substrate. A total of 529 elements are placed on a 1.575 mm substrate $\left(6.5\lambda_{o}\times 6.5\lambda_{o}\right)$. The inter-element spacing in the array is kept at $0.3\lambda_{o}$ for equal spacing and to avoid the unnecessary coupling of the HSS elements. Grating lobes are avoided by maintaining the spacing between the elements at less than $0.5\lambda_{o}$ [41]. As reflectarrays are spatially fed, the incident phase originates from different paths from the feed, which are adjusted locally. The phase compensation of the incident field is performed for each phasing element using Equation (1):

(1) $$\varphi \left(x_{i},y_{j}\right)=-K_{o}\left(\sin\left(\theta_{b}\right)\cos\left(\phi_{b}\right)x_{i}-\sin\left(\theta_{b}\right)\sin\left(\phi_{b}\right)y_{j}\right)$$

where $K_{o}$ denotes the propagation constant in vacuum, and $x_{i},y_{i}$ represent the coordinates of the element *i*. To introduce a phase shift into each element of the reflectarray, the difference between (1) and the phase of the incoming field from the feed must be obtained as follows:

(2) $$\varphi_{Ri}=K_{o}\left(d_{i}-\left(x_{i}\cos\phi_{b}+y_{i}\sin\phi_{b}\right)\sin\theta_{b}\right)$$

where $d_{i}$ represents the distance between the phase center of the feed and the element. The phase shift is adjusted to match the phase. The phase difference in each unit cell is obtained using Equations (1) and (2), as shown in Figure 7. The elements are arranged with respect to rows, and columns are placed on the reflectarray panel to obtain a progressive phase based on the phase of each component. Figure 3 shows the phase that provides the parameters of the HSS patch for the phase distribution. The dimensions of a unit cell play a crucial role in the design of a reflectarray. A more than $360^{\circ }$ phase range corresponds to a unit cell. The equations above were used to obtain the phase distribution of the reflectarray aperture. The mapping of the unit cell along the aperture is in accordance with the phase range values. The spillover and illumination efficiency must also be taken into account, as they are critical factors in the performance of reflectarrays. By performing a phase distribution arrangement, we can minimize its effects on the performance of the reflectarrays in practical scenarios.

**Figure 8:**
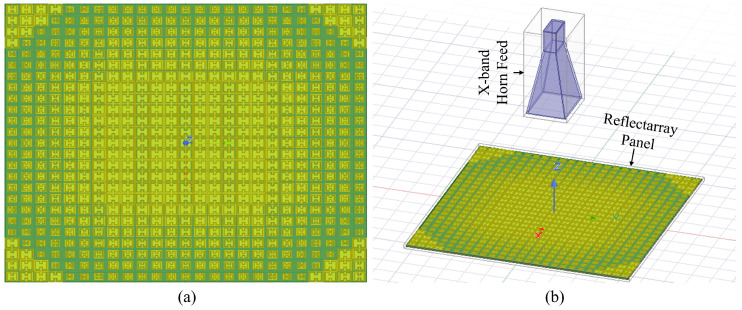
A relectarray with a feed horn in a (hybrid) FEBI method. (**a**) Schematics of array. (**b**) Horn antenna with the panel.

A schematic of the designed reflectarray is shown in Figure 8a. The proposed reflectarray has a $6.5\lambda_{o}\times 6.5\lambda_{o}$ aperture size, which requires considerable computational resources and time for simulation. To reduce simulation time, a hybrid method based on a Finite Element Boundary Integral (FEBI) algorithm is utilized. The Finite Element Method and Integral Method are combined in this algorithm [42]. Electrically large structures and complex systems can be handled easily using this algorithm. By using the hybrid FEBI method, the far-field characteristics of the reflectarray are achieved. A 20-core Dell workstation with a workable memory of 128 GB is used for the full reflectarray simulations. The GPU required approximately 15 h to fully simulate the reflectarray. This is because as the array size increases, the computational complexity grows significantly due to the need to model and simulate a larger number of elements, each with precise control and element interaction. This increase in complexity necessitates not only more powerful computational resources but also more efficient algorithms to handle the increased data processing requirements. For larger or more complex arrays, parallel processing techniques and the use of high-performance computing (HPC) environments could be essential to manage the simulations within a reasonable timeframe. Furthermore, we discuss the potential for algorithmic optimization, such as the use of fast Fourier transform (FFT)-based methods or adaptive meshing techniques, which can reduce computational load while maintaining accuracy.

Figure 8b shows the FEBI system with the reflectarray panel and feed. A center-fed reflectarray within FEBI boundaries is designed. The F/D ratio represents the ratio of the focal length to the diameter of the reflectarray. This is important because an optimal value must be selected to improve illumination efficiency and reduce spillover losses. In this study, an optimized value of 1 is selected for the F/D ratio to obtain the maximum gain and aperture efficiency.

## 4. Results and Discussion

**Figure 9:**
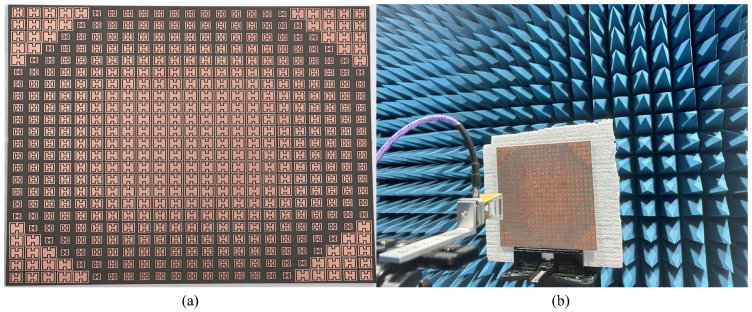
Schematics of the fabricated prototype. (**a**) Reflectarray panel. (**b**) Testing setup of the fabricated reflectarray.

A prototype of the simulated reflectarray is fabricated on a high-quality thin Rogers 5880 substrate. The fabricated reflectarray is a compact single-layer design with no air gaps. Chemical etching is used to fabricate the radiating array elements. It is a widely used process in the fabrication of radiating array elements for reflectarrays and other antenna systems. This method involves using chemical solutions to selectively remove material from a substrate to create the desired pattern of the array elements. Post-processing of the fabricated reflectarray was performed to remove any residuals so that optimal operation could be obtained. The schematics of the fabricated reflectarray are shown in Figure 9a, and the measurement setup in an anechoic chamber is illustrated in Figure 9b.

The far-field measurement setup is used to obtain the radiation pattern and gain of the proposed reflectarray. There are slight discrepancies between the fabricated and simulated results, which are caused by the errors introduced during fabrication and during measurement in the anechoic chamber. The reflectarray and feed horn are rotated $90^{\circ }$ to achieve a radiation pattern for both the *E* and *H*-planes. A DRH67 double-ridged horn antenna is used because of its good operation in terms of high bandwidths, high gain, and precision. The boundary conditions used the same feed characteristics, ensuring that the surrounding environment was controlled to minimize external interference. The physical dimensions of the reflectarray and the placement of the feed antenna mirrored the simulation setup. The dielectric constant and tangent of the Rogers 5880 substrate were carefully measured and matched to the values used during simulations. Similarly, the calibration of the instruments was performed to minimize errors in order to accurately capture the reflectarray performance.

**Figure 10:**
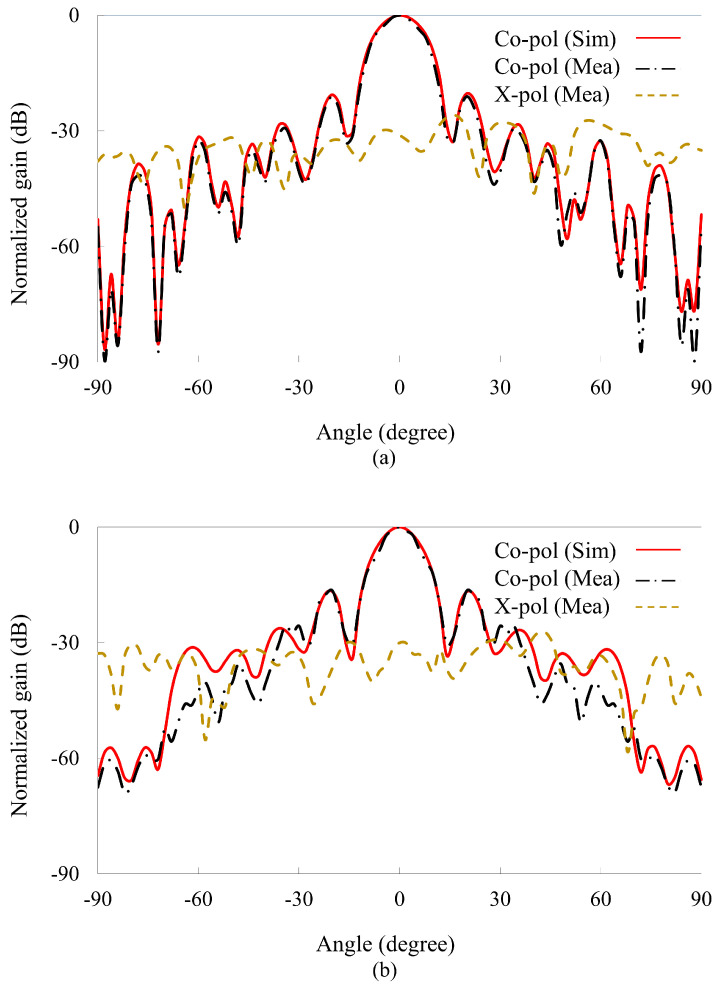
Normalized radiation patterns at 10 GHz. (**a**) E-plane. (**b**) H-plane.

Figure 10 illustrates the normalized radiation patterns, both simulated and measured in the far field for the *E*- and *H*-planes. The radiation patterns for the X-band in the principal planes are recorded at a center frequency of 10 GHz. It can be observed that the main beam lobe is set at $0^{\circ }$ for the center-fed reflectarray. Moreover, the recorded SLLs and cross-polarization levels are less than −21 dB and −33 dB. It is shown in the results that in both the *E* and *H*-planes the main lobe is at $0^{\circ }$, aiming at the center. The patterns experience a modest deterioration when the SLLs and cross-polarization levels increase owing to fabrication errors. The precise placing of the reflectarray elements on the substrate minimizes the side lobe levels, which were instrumental in reducing the cross-polarization. Additionally, the orientation and geometry of the H-shaped slotted patch were optimized to ensure that unwanted polarization components were minimized. Owing to the two-fold symmetric element and y-polarized incidence, the reflectarray exhibits low SLLs and cross-polarization.

**Figure 11:**
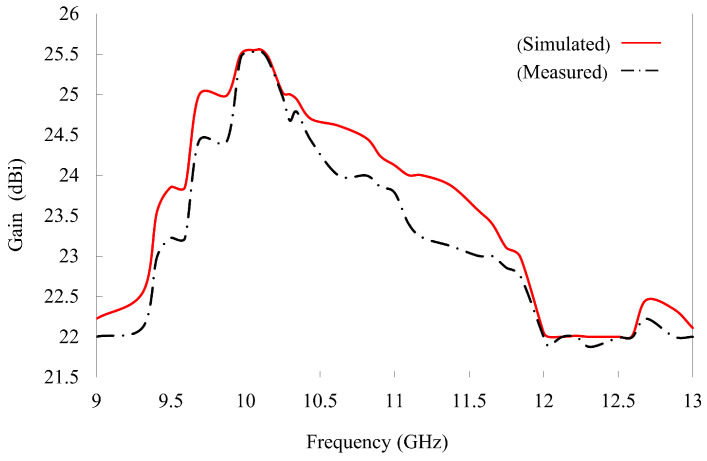
The realized gain of the reflectarray.

The reflectarray provides a maximum gain of 25.5 dBi at a center frequency of 10 GHz, as shown in Figure 11. Thus, a broad 1 dB gain bandwidth of 20% is achieved. The antenna aperture efficiency is calculated from the realized gain (G) and the physical aperture area (A) using $\left(\eta_{a}=G_{abs}\lambda^{2}/4\pi A\right)$. The maximum aperture efficiency reached 63.7% despite the presence of reflection loss (0.43 dB) (Figure 6b) owing to the loss tangent (tanδ=0.0009 of the Rogers 5880 substrate. The factors limiting the overall efficiency include element losses, phase quantization errors, edge effects, and feed illumination. Optimizing the HSS patch, increasing the number of phase states, implementing edge treatments, and improving feed design could further enhance the efficiency of the designed reflectarray. A performance comparison of our designed reflectarray with previous studies is presented in Table 2. Our designed reflectarray performed well in the X-band and no additional air layer was required. From Table 2, it can also be seen that our proposed reflectarray can provide a good trade-off between the 1 dB gain bandwidth, peak gain, aperture efficiency, SLLs, and cross-polarization level.

## 5. Conclusions

In this study, an X-band single-layer HSS patch broadband reflectarray is designed. The HSS slotted patch is designed to achieve linearity and additional resonance for a wider phase range exceeding $480^{\circ }$. The phase range is varied by changing the length of the embedded slots within the patch. A high-quality thin Rogers 5880 substrate with a thickness of 1.575 mm is optimized with a ground layer to obtain a smoother phase response. A reflectarray is simulated using the FEBI method and then fabricated and tested in an anechoic chamber. A maximum gain of 25.5 dBi and an aperture efficiency of 63.7% are obtained for a center frequency of 10 GHz. Additionally, a 1-dB gain bandwidth of 20% is realized for the X-band. The HSS patch is a suitable candidate for X-band applications based on the simulated and measured results. This design can be used in satellite communication as reflectors in the CubeSat for high gain, directive beams with reduced weights and cost compared to traditional parabolic reflectors or phased arrays. Additionally, the precise beam steering capabilities of reflectarrays are beneficial in radar systems, enabling improved target detection and tracking. We will also suggest future research directions to build on the current study. Exploring multilayer designs could further enhance the reflectarray’s bandwidth and efficiency by allowing more complex phase control and reducing losses. 

## Figures and Tables

**Table 1:** The parameters of the designed HSS patch unit cell.

Parameter	P	L	$L_{1}$	$w_{1}$	$L_{2},w_{2}$
Values	9 mm	8–4.4 mm	6.4–2.4 mm	0.5 mm	2 mm

**Table 2:** Comparison with previously reported reflectarrays.

References	This Work	[27]	[28]	[29]
Center frequency (GHz)	10	10	10	10
Gain (dBi)	25.5 dBi	24 dBi	25 dBi	22.8 dBi
Aperture efficiency (%)	63.7%	48%	52.8%	32%
1 dB Bandwidth (%)	20%	18%	19.5%	-
Side lobe levels (SLLs)	−21	−22	−22	−14
X-pol (dB)	−33	−35	−35	−25
Air layer	No	Yes	Yes	No

## Data Availability

The data can be provided upon request from the authors.
